# Impact of temporal variability of radon concentration in workplaces on the actual radon exposure during working hours

**DOI:** 10.1038/s41598-021-96207-9

**Published:** 2021-08-20

**Authors:** G. Venoso, A. Iacoponi, G. Pratesi, M. Guazzini, L. Boccini, E. Corbani, S. Bucci, F. Leonardi, R. Trevisi, M. Ampollini, S. Antignani, M. Caprio, C. Carpentieri, C. Di Carlo, F. Bochicchio

**Affiliations:** 1 National Center for Radiation Protection and Computational Physics (Italian National Institute of Health), Viale Regina Elena, 299 Rome, Italy; 2 Florence Section, Regional Agency for Environmental Protection in Tuscany (ARPAT), Via Ponte alle Mosse 211, 50144 Florence, Italy; 3 Italian National Institute for Insurance against Accidents at Work (INAIL)-DiMEILA, Via Fontana Candida, 1 Monte Porzio Catone, Rome, RM Italy

**Keywords:** Health occupations, Risk factors

## Abstract

For workplaces where significant diurnal variations in radon concentrations are likely, measurements to evaluate average radon concentration during working hours could be useful for planning an optimized protection of workers according to the 2013/59/Euratom Directive. However, very few studies on this subject, generally limited to periods of few weeks, have been published. Therefore, a study has been conducted to evaluate the actual long-term radon exposure during working hours for a sample of 33 workplaces of four different types (postal offices, shops, restaurants, municipal offices), mainly located at the ground floor, and with expected considerable air exchange rate occurring during working hours due to frequent entrance/exit of persons or mechanical ventilation. The results show that the difference between the average radon level during working hours and that one during the whole day is about 20% on average and ranges from 0 to 50%. These observed differences, generally smaller compared with those found in other similar studies, are nearly the same if the analysis is restricted to workplaces with annual radon level higher than 300 Bq m^–3^, and therefore natural or mechanical ventilation normally present during working hours of the monitored workplaces cannot be considered an effective mitigation measure. However, the costs and time-response characteristics of the active monitors, as those used for the present study, will probably allow using more frequently a similar measurement strategy in workplaces.

## Introduction

The new international regulations and recommendations^1,2^ have introduced the new concept of reference level (RL). They require that, for workplaces resulted to have an annual average radon level higher than RL, action has to be taken in agreement with the optimization principle to reduce radon exposure. Besides mitigation measures, the optimization actions may also include measurements to investigate the activity concentration during working hours, if appropriate^3^. Indeed, in some workplaces may occur that radon concentrations averaged during the working hours were much lower (well below a RL of 300 Bq m^–3^) than those averaged over the whole day, in some cases^4,5^ also higher than 1000 Bq m^–3^. It is the case of some workplaces or schools having mechanical ventilation systems active during the working hours only, being turned off during night-time and the weekends.

In some countries, such as Norway^6^, Canada^7^ and Finland^8^, for these types of workplaces, the following monitoring strategy is adopted to demonstrate compliance with reference levels: if the results of long-term radon concentration measurements carried out with passive devices were higher than the national reference level, continuous monitoring of at least one week is recommended or required. However, in literature few experimental studies exist investigating the difference between the average radon level during working hours and the yearly averaged concentration. Moreover, to the authors' knowledge, no extensive study has been performed over periods longer than few weeks.

In the framework of a project promoted by INAIL, four types of workplaces have been identified among those for which it is likely to find significant diurnal variations in radon concentrations. In a sample of these workplaces, a measurement approach based on long-term radon monitoring over periods of 6–12 months, using both passive and active radon devices simultaneously, has been adopted. The aim of this study was to find if this protocol, suitable to allow the evaluation of the average radon concentration during only the actual working hours, can be employed in this kind of workplaces using an affordable active device recently introduced on the market.

## Materials and methods

### Sampling

Four types of workplaces have been identified among those ones with public access (Table 1) and for which different radon levels are expected to occur during the working hours due to a likely increase of natural or mechanical ventilation. These workplaces were mainly located in Municipalities classified as *radon prone areas* in Tuscany and preferentially among those resulted to have an average annual radon concentration higher than 150 Bq m^–3^ in the framework of a radon survey conducted in this region one decade ago^9^.

**Table 1:** Workplaces chosen for the study.

Type of workplaces	No. of buildings	No. of rooms
Postal offices	5	8
Shops	5	5
Restaurants	6	10
Municipal offices	6	10
Total	22	33

For each workplace, rooms to be monitored were chosen among those having ventilation conditions representative of nearly all the rooms. In presence of rooms with different ventilation regimes, if possible, another active device has been deployed.

Most of the rooms (85% of them) were located at the ground floor or basement, i.e., at floors where radon concentration measurements are mandatory for workplaces within *radon priority areas* of the EU Member States*,* according to the current European Directive^2^.

### Experimental protocol

In each room of the chosen workplaces, annual radon concentration measurements were performed using passive radon devices over two consecutive 6-months periods. With the aim to estimate radon exposure during working hours, active radon detectors were simultaneously deployed with passive radon devices during the first 6-month period.

Although a 6-month period (occurring mainly in winter and spring seasons) was considered adequate to estimate the impact of air exchange rate occurring during working hours, in a sample of 11 rooms, active radon detectors were exposed also during the second 6-month period. These rooms were prevalently chosen among those resulted to have average radon concentration during the first 6-month period higher than 150 Bq m^–3^, according to the results of active radon detectors.

The first 6-month period started in winter (between December 2018 and February 2019) and ended in summer (between June and August 2019). The annual exposure ended between December 2019 and February 2020.

For each room and each exposure period, for the active radon device an acquisition time of 1 h was chosen, which allow to have an adequate time-resolution for estimating the radon concentration during the working hours^10^.

### Radon devices

Passive devices were alpha track detectors (ATD), having CR-39 (TASL) as sensitive material, enclosed in a small diffusion chamber. Etching was performed in 6.25 NaOH solution for 60 min and track counting was performed with a fully automated image analysis system (TASLIMAGE).

Measurements have been conducted in the Radioactivity laboratory of ARPA Toscana which was accredited on the basis of ISO/IEC 17025^11^.

As active devices, continuous monitors based on the electrostatic collection of the radon daughter emitting alpha particles on a silicon detector (model TSR 4 M produced by TESLA, Czech Republic). These devices allow to detect radon concentration every hour and they have a nominal sensitivity of about 25 counts per hour (*cph*) at a mean radon concentration of 100 Bq m^–3^. Therefore, they have an uncertainty of about 12% at 300 Bq m^–3^ due to the random Poisson component. The minimum detectable activity concentration is about 100 Bq m^–3^ for 1- h measurements.

These devices are also dependent on internal temperature and humidity which affect the electrostatic collection of the charged radon daughters on the detectors. Therefore, both these climatic parameters have been detected by the instrument and the sensitivity corrected accordingly.

The calibration of these devices has been performed by the means of an ad hoc intercomparison in our lab using professional *AlphaGUARD* as reference monitors.

## Results

### Intercomparison between AlphaGUARD and active monitors used for the study

Before the starting of the survey, all the active radon devices were deployed along with a professional radon monitor (AlphaGUARD PQ2000PRO) in order to evaluate their accuracy and their time response. This intercomparison was performed in an office room having an average radon level of about 200 Bq m^–3^, but normally experiencing day-night radon concentration variation, over a period of about 10 days.

**Figure 1:**
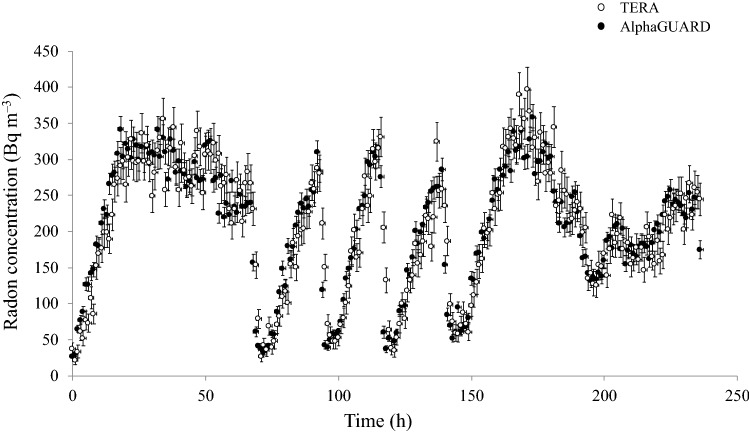
Intercomparison between AlphaGUARD PQ2000PRO (in diffusion mode) and TERA TSR 4 M active device exposed in a room for about 10 days. An acquisition time of 1 h was chosen for both the instruments. The uncertainties reported in the graph have a coverage factor k equal to 1.

It was found that on average the calibration factor of these probes was 50% lower than that of the AlphaGUARD. Instead, the time response of these probes resulted to be as good as that of the professional monitor (see Fig. 1).

Notably, it is a good time-response of the active devices that allow to correctly evaluate any difference of radon levels during working hours as compared with the remaining ones. Therefore, the lack of accuracy was considered not important considering the aim of this study. However, all the calibration factors of the TERA probes were corrected taking into account the average radon concentration measured by the AlphaGUARD monitor, and the following analyses have been performed using the corrected calibration factors.

### Comparison between active and passive devices

For each monitored room, average radon concentration results were reported in Table 2 for each exposure period. In this table, both results of the passive devices (RnC_CR-39_) and active devices are included. For the active devices, radon levels were integrated over all the days (RnC_H24_) and over the working hours (RnC_WH_) and the ratios of these values (R_WH_/_h24_) are also reported.

**Table 2:** Results of the average radon concentration measurements (over two 6-month periods and over 1 year) performed using passive radon devices (RnC_CR-39_) and active ones integrating radon levels over the all the days (RnC_H24_) and over the working hours (RnC_WH_).

Type of workplace	Building	Room	Room type	Floor	Access to the public	1st 6-month period				2nd 6-month period				12-month period			
						RnC_CR-39_ (Bq m^–3^)	RnC_H24_ (Bq m^–3^)	RnC_WH_ (Bq m^–3^)	R_WH_/_h24_	RnC_CR-39_ (Bq m^–3^)	RnC_H24_ (Bq m^–3^)	RnC_WH_ (Bq m^–3^)	R_WH_/_h24_	RnC_CR-39_ (Bq m^–3^)	RnC_H24_ (Bq m^–3^)	RnC_WH_ (Bq m^–3^)	R_WH_/_h24_
Postal office	1	1	Office	0		28	38	29	0.8	40				34			
	2	1	Office	0		17	12	9	0.7	17				17			
	3	1	Counter room	1	Yes	205	167	144	0.9	249	183	166	0.9	228	175	155	0.9
	3	2	Office	1		227	183	161	0.9	246				237			
	4	1	Storage area	0		161	137	110	0.8	172				167			
	4	2	Counter room	0	Yes	171	168	137	0.8	204				188			
	5	1	Counter room	0	Yes	414	645	580	0.9	432	700	731	1.0	423	673	656	1.0
	5	2	Office	0		398	383	368	1.0	440				420			
Restaurant	1	1	Restaurant	0	Yes	181	279	258	0.9								
	2	1	Kitchen	0		33	45	38	0.8	43				38			
	2	2	Restaurant	0	Yes	64	57	52	0.9	72				68			
	3	1	Kitchen	0		54	91	73	0.8	78				66			
	3	2	Restaurant	0	Yes	159	125	114	0.9	174				167			
	4	1	Kitchen	− 0.5		67	78	65	0.8	76				72			
	4	2	Restaurant	− 1	Yes	180	225	203	0.9	160	269	237	0.9	170	248	220	0.9
	5	1	Kitchen	1		3490	4621	3114	0.7	4209	5744	4695	0.8	3864	5205	3937	0.8
	5	2	Restaurant	0	Yes	1159	1105	1015	0.9	1446	1716	1656	1.0	1308	1423	1349	0.9
	6	1	Restaurant	0	Yes	246	134	132	1.0	204	131	132	1.0	225	132	132	1.0
Shop	1	1	Sales area	0	Yes	382	411	221	0.5	284				332			
	2	1	Sales area	0	Yes	201	61	40	0.7	168				184			
	3	1	Sales area	0	Yes	173	153	129	0.8	194				184			
	4	1	Sales area	0	Yes	215	114	84	0.7	254				235			
	5	1	Sales area	0	Yes	117	82	70	0.9	366				244			
Municipal office	1	1	Office	1	Yes	314	278	210	0.8	308				311			
	1	2	Police office	1	Yes	326	314	197	0.6	383				355			
	2	1	Police office	0	Yes	420	376	291	0.8	477	567	462	0.8	449	473	377	0.8
	2	2	Archive room	0		566	526	418	0.8	619	682	568	0.8	593	605	494	0.8
	3	1	Archive room	− 0.5		90	92	74	0.8	86				88			
	3	2	Office	0		202	163	94	0.6	204				203			
	4	1	Office	0		458	446	318	0.7	636	566	482	0.9	548	507	401	0.8
	5	1	Office	0		384	279	247	0.9	415	373	336	0.9	400	327	292	0.9
	5	2	Office	0	Yes	206	134	101	0.8	230	236	186	0.8	218	186	144	0.8
	6	1	Office	0	Yes	299	298	268	0.9	299				299			

The uncertainties for RnC_H24_ and RnC_WH_ (not reported in the table) are of the order of 10% (k = 1).

Legends for floor: 0 = ground floor; − 0.5 = basement; 1 = first floor.

As shown in Table 2, for each type of workplaces there is at least one room with annual average radon concentrations higher than 300 Bq m^–3^, the maximum reference level for a European Member State according to the 2013/59/Euratom Directive^2^. This was expected considering that in the sampling strategy workplaces were preferably chosen in Municipalities classified as *radon prone areas* in 2012.

**Figure 2:**
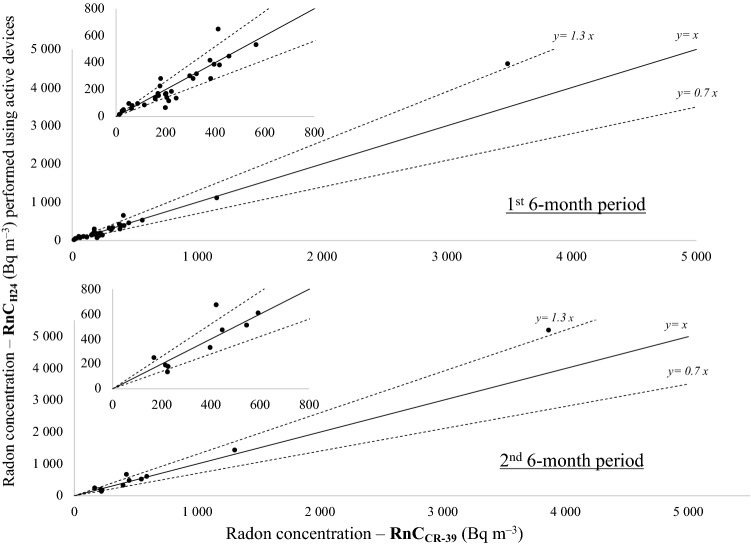
Comparison between average radon concentrations results measured using passive radon devices (x-axes) and active devices (y-axes) both for first (above) and the second 6-month period of exposure (below). The smaller plots are a zoom in the range 0–800 Bq m^–3^.

Results of the average radon measurements performed using active radon devices (RnC_H24_ in Table 2) showed enough agreement with those performed using passive radon devices (RnC_CR39_ in Table 2). Their differences are mostly lower than 30% (the area between the two dashed lines in Fig. 2) especially for rooms having a mean radon level higher than 300 Bq m^– 3^. In particular, for the first 6-month period, the median of the difference between active and passive devices is equal to 7% (range: − 70% to 70%), but for radon level higher than 300 Bq m^–3^, the median is 4% (range: − 40% to 30%).

Regarding passive devices, factors that might have had an impact on the increase of uncertainties could be the ageing and fading^12,13^ as well as the presence of thoron^14^ and, for very high radon level, the tracks overlapping^15^. Regarding active devices, besides thoron^16^, another possible influencing factor on their response could be a not correct characterization as a function of climatic conditions since the used monitor are highly dependent on temperature and humidity^17^.

These results—although not as good as those of metrological type carried out in a radon chamber—are similar in terms of accuracy to those performed in the framework of intercomparison under field conditions^18,19^.

### Radon concentration during working hours (RnC_WH_) versus during the total time (RnC_H24_)

**Figure 3:**
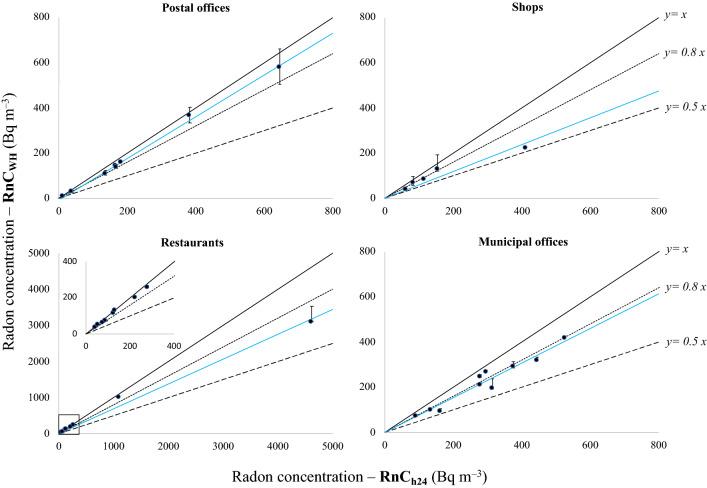
Comparison between average radon concentrations, over the all the days and hours (RnC_H24_) and over the working hours (RnC_WH_) only, for the first 6-month period of exposure. The full lines represent the bisecting lines. The dotted and dashed lines represent lines with slopes of 0.8 and 0.5, respectively. The best fit lines are in blue. As y error bars are reported the maximum differences with RnC_WH_ (reported in Table 3) estimated varying of 1–2 h the actual working hours. The smaller plot in the *restaurants* box is a zoom in the range 0–400 Bq m^–3^.

The average radon level over the working hours (RnC_WH_) generally resulted lower than the average over all the hours (RnC_H24_): this difference is always not higher than 50% and in most cases is not higher than 20% for each type of workplaces (see Table 2 and Fig. 3).

In particular, for the first 6-month period of exposure, the average R_WH_/_h24_ is 0.8 (range: 0.5–1.0). For the 11 rooms with also the second 6-month of exposure, the values of R_WH_/_h24_ are even (slightly) higher (see Table 2) being the average equal to 0.9 (range: 0.8–1.0).

Moreover, on average there are no significant differences, in terms of R_WH_/_h24_, between rooms with access to the public and the remaining ones. Similarly, no differences are found between rooms with radon level higher and lower than 300 Bq m^–3^.

In order to find if these results could be affected by variation of the actual working hours, a sensitivity analysis was performed varying the working hours: for each room, opening (and closure) times were shifted of both 1 and 2 h and the average radon concentration during the working hours (RnC_WH_) was calculated accordingly (see Table 3).

**Table 3:** Sensitivity analysis: differences with the average radon levels during working hours (RnC_WH_) as reported in Table 1 and average radon levels during working hours shifting the opening hours of 1 and 2 h (both back and forth).

Type of workplace	Building	Room	RnC_WH_ (Bq m^–3^)	Difference with RnC_WH_ of							
				RnC_WH_ (− 2 h)		RnC_WH_ (− 1 h)		RnC_WH_ (+ 1 h)		RnC_WH_ (+ 2 h)	
				(Bq m^–3^)	%	(Bq m^–3^)	%	(Bq m^–3^)	%	(Bq m^–3^)	%
Postal office	1	1	29	5	16	2	8	− 2	− 8	− 4	− 12
	2	1	9	2	22	1	14	− 1	− 9	− 1	− 12
	3	1	144	3	2	2	1	− 1	− 1	− 2	− 1
	3	2	161	3	2	1	1	− 1	− 1	− 3	− 2
	4	1	110	13	11	6	5	− 5	− 4	− 7	− 6
	4	2	137	14	10	6	5	− 4	− 3	− 5	− 4
	5	1	580	81	14	41	7	− 41	− 7	− 75	− 13
	5	2	368	34	9	18	5	− 17	− 5	− 33	− 9
Restaurant	1	1	258	7	3	2	1	− 3	− 1	− 1	0
	2	1	38	− 1	− 3	− 1	− 2	− 1	− 1	1	2
	2	2	52	− 1	− 3	− 1	− 2	0	0	2	4
	3	1	73	− 2	− 3	− 1	− 2	0	0	3	4
	3	2	114	− 4	− 3	− 2	− 2	1	1	2	2
	4	1	65	− 1	− 2	− 1	− 2	2	2	6	9
	4	2	203	6	3	3	1	0	0	6	3
	5	1	3114	414	13	123	4	11	0	274	9
	5	2	1015	48	5	20	2	− 9	− 1	3	0
	6	1	132	3	2	2	1	0	0	− 2	− 2
Shop	1	1	221	64	29	31	14	− 10	− 4	10	5
	2	1	40	4	10	1	3	1	2	4	10
	3	1	129	27	21	12	10	− 9	− 7	− 12	− 10
	4	1	84	28	33	14	17	− 12	− 14	− 16	− 19
	5	1	70	7	10	4	6	− 4	− 5	− 5	− 7
Municipal office	1	1	210	8	4	6	3	0	0	6	3
	1	2	197	41	21	17	9	− 6	− 3	− 1	0
	2	1	291	21	7	10	3	− 4	− 2	− 5	− 2
	2	2	418	9	2	3	1	2	0	8	2
	3	1	74	− 1	− 1	0	0	2	2	4	5
	3	2	94	10	11	4	5	1	1	8	8
	4	1	318	17	5	8	2	1	0	9	3
	5	1	247	9	4	5	2	− 3	− 1	− 2	− 1
	5	2	101	8	8	7	7	− 4	− 4	− 4	− 4
	6	1	268	26	10	11	4	− 10	− 4	− 18	− 7

The results of this sensitivity analysis show that the impact of changing the working hours is generally non-significant. The RnC_WH_ varies on average by 2% and at maximum by 33%. For those rooms with average radon concentrations higher than 300 Bq m^–3^, the impact is even lower: RnC_WH_ varies on average by 2% at maximum by 14%.

This analysis confirms that, as compared to average results over all the time (including nights and weekends), radon reduction during working hours usually resulted not higher than 20% for the type of workplaces considered in this study. Results of the present study indicate that the effect of natural or mechanical ventilation during the working hours of the monitored workplaces cannot be considered as an effective mitigation measure, and in turn to avoid the implementation of remedial actions in case annual average radon concentration RnC_H24_ resulted to be higher than a reference level (RL). It is worth noting that some of these workplaces, having RnC_H24_ slightly higher than a reference level (RL), could have instead a RnC_WH_ lower than RL, even if close to it. In these cases, some measures aimed to reduce radon levels still needed to be performed, considering that the new concept of reference level require that optimization (and eventually radon exposure reduction) has to be implemented with priority above RL, but should also be implemented below it^20^.

## Discussion

Few studies were carried out to evaluate the effect of the ventilation during the working hours on radon levels, generally finding a much higher radon reduction during the working hours compared with the present study. In the UK, in a retail shop, a reduction of about 75% starting from an average radon concentration of about 1400 Bq m^–3^ was attributed to the turning on of an air-conditioning system^21^. A similar reduction during working hours (about 70%) was found in two schools in Hungary, starting from about 500 and 300 Bq m^–3^ ^22^.

In Nordic Countries a very high reduction (higher than 90%) was found in two schools and public buildings. In particular, in Finland radon decrease from about 2000 Bq m^–3^ to about 100 and 20 Bq m^–3^, in a school and a day-care centre, respectively^5^. In Norway, in a seminar room of a university, average radon level decreased from about 5000 Bq m^–3^ to 130 Bq m^–3^, and, in an elementary school, from 300 Bq m^–3^ to 20 Bq m^–3^ ^4^.

Notably, the above-mentioned studies were performed over periods not longer than 1–2 weeks, thus they were not able to evaluate the stability of the reduction during the working hours over longer periods, i.e., some months or a year, as it was done in the present study.

**Figure 4:**
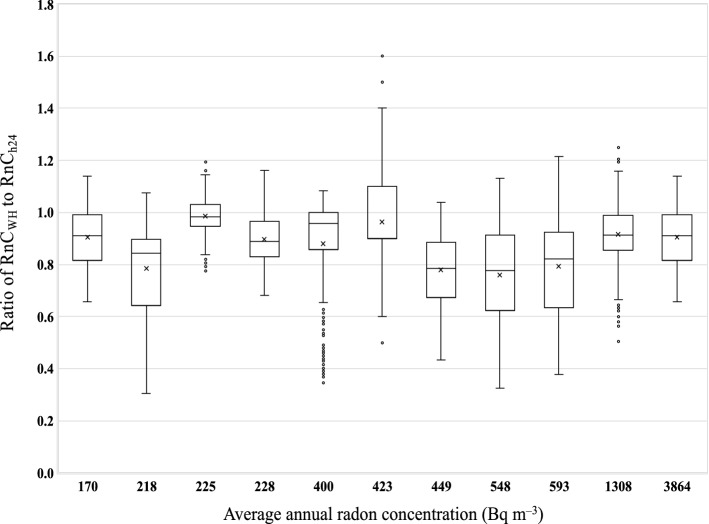
Box-plot of the ratio between RnC_WH_ and RnC_H24_ estimated for each 7-day period of the year to the 11 rooms for which annual radon measurements with active devices were performed. On the x-axis, the average annual radon concentrations for these 11 rooms are reported.

In fact, for each of the 11 rooms for which annual radon concentration measurements were performed using active devices, RnC_WH_ and RnC_H24_ were calculated for each 7-day period of the year. As shown in Fig. 4, the interquartile ranges of R_WH_/_h24_ are from about 0.6 to 1.1, with a minimum value of about 0.3, and maximum value of 1.6.

**Figure 5:**
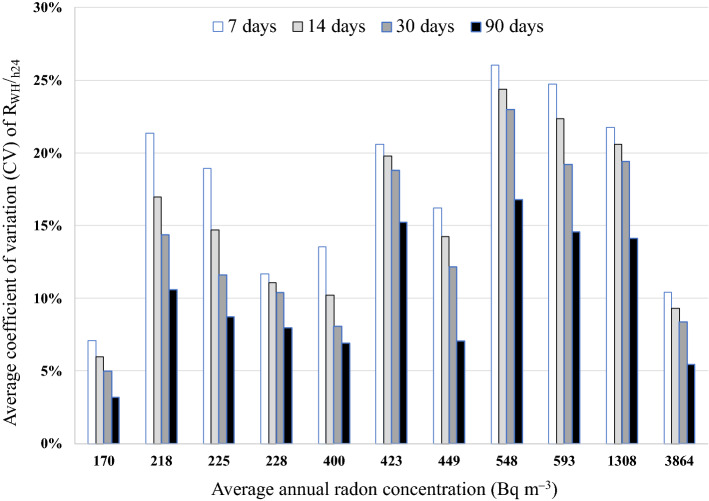
Average coefficient of variation of the ratio between RnC_WH_ and RnC_H24_ (R_WH_/_h24_) estimated for each 7-day, 14- day, 30-day, and 90-day period of the year for the 11 rooms for which annual radon measurements with active devices were performed. On the x-axis, the average annual radon concentrations for these 11 rooms are reported.

The choice of a random 7-day period over the year to estimate R_WH_/_h24_ introduces an uncertainty—in terms of coefficient of variation (CV) of R_WH_/_h24_—of 17% on average (range: 7%-26%). As shown in Fig. 5, the uncertainty obviously decreases (but not with the same rate for all the rooms) as the number of days chosen for the estimation of R_WH_/_h24_ increases. In particular, the CV is 14% and 10% on average for a period of 30 and 90 days, respectively.

These results suggest to choose a period of several months in order to properly estimate using active radon monitors the impact of ventilation during working hours on the average annual radon concentration. However, further investigations are needed to confirm these results also on workplaces experiencing a radon reduction during the working hours much higher than that found in the present study.

Considering the costs of the active monitors recently introduced on the market, like those used for the present study, the choice to continuously measure radon concentration over a period of one year will probably be a good option for some types of workplaces. These types of active monitor could also be adopted as time-resolved personal measurement devices for exposed workers, i.e., in those workplaces where the *exposure of workers is liable to exceed an effective dose of 6 mSv per year or a corresponding time-integrated radon*, as reported in art. 35.2 of 2013/59/Euratom Directive^2^. Up to now, in these cases (e.g., caves or mines), the evaluation of the effective radon exposure during working hours was generally performed using passive radon devices wore by the workers during the working hours and stored, when not in use, in low radon environments (Shahrokhi et al., 2017) or by passive radon monitors sensitive to radon only during the working hours^23–25^. However, time-resolved measurements performed using active monitors are in principle far more informative than the integrating measurement using passive devices, and the huge reduction of the costs the active devices will probably increase their use also as for personal radon exposure assessment.

Nevertheless, the results of the comparison of a type of active devices with passive ones carried out in the present study, suggest to perform further investigation in order to study their performances (accuracy, thoron interference, stability, and so on) both in radon chamber and under field conditions with exposure time of several months. Until then, it is however preferable to use, as in the present study, a passive device in combination with an active device having a good time-response: the first one to correctly evaluate the long-term average radon concentration; the second one to evaluate the ratio between radon levels during working hours as compared with the remaining ones and to correct the long-term average accordingly.

## Conclusions

In the present study, active radon devices were used to evaluate the impact of the increased ventilation during working hours on the actual radon exposure of workers in 33 workplaces of different types (postal offices, shops, municipal offices, and restaurants).

The average difference between the average radon levels during working hours and during the whole days (including nights and weekends) resulted to be about 20%, and ranged from 0 to 50%. These results—equal also if the analysis is restricted to workplaces having average radon level higher than 300 Bq m^–^—suggest that natural or mechanical ventilation during the working hours of the monitored workplaces cannot be considered as an effective mitigation measure.

However, the costs of the active monitors as those used for the present study, as well as their good time-response characteristics, will probably lead to use them more frequently for workplaces where significant diurnal/nocturnal variations in radon concentrations are likely.
